# Factors Influencing Renovation Decisions By Care Facility Operators: An Investigative Study in Australia

**DOI:** 10.1093/geroni/igaf122.3195

**Published:** 2025-12-31

**Authors:** Si Chen

**Affiliations:** Bond University, Southport, Queensland, Australia

## Abstract

A supportive built environment is essential in residential care facilities for individuals with long-term care needs, both physically and emotionally. However, upgrading existing facilities presents various challenges. Before bridging the gap between theoretical and practical needs in supportive built environment adaptation, it is crucial to first understand the reasons behind renovation decisions from the perspective of care facility operators, a topic that has received limited attention in the past. This study aims to identify the key drivers of renovation decisions and explore the interrelationships between supportive design, renovation project management, and the decision-making process. Interviews were conducted with professionals from three major stakeholder groups: designers, project managers and consultants, and care facility operators. Participants were selected based on their involvement in past or current renovation projects in residential care facilities in Australia. Inductive analysis of interview transcripts identified three main themes: (1) information to guide renovation decisions, (2) decision-making efficiency, and (3) information mismatch. The study found three primary drivers of renovation decisions: budget, regulatory compliance, and operational and workflow efficiency. While some findings did not directly address the research questions, they provide valuable insights into the challenges and decision-making processes in residential care facility renovations. This research contributes to the development of practical tools and frameworks to enhance decision-making and project execution. Additionally, it offers insights from a broader group of stakeholders, informing more effective and strategic renovation processes in residential care facilities.

